# Supplementary material for: A cell atlas of adult muscle precursors uncovers early events in fibre‐type divergence in Drosophila
Source: EMBO Rep. 2020 Aug 19;21(10):e49555. doi: 10.15252/embr.201949555 (PMC7534622; doi:10.15252/embr.201949555)
Supplement: Supplementary file 1 — Appendix [file EMBR-21-e49555-s001.pdf]

Appendix

Table of Content

Appendix Figures *2*

Appendix Figure S1 2

Appendix Figure S2 4

Appendix Figure S3 6

Appendix Figure S4 7

Appendix Tables *8*

Appendix Table S1 10

Appendix Table S2 11

Appendix Table S3 14

Appendix Table S4 15

Appendix Figures

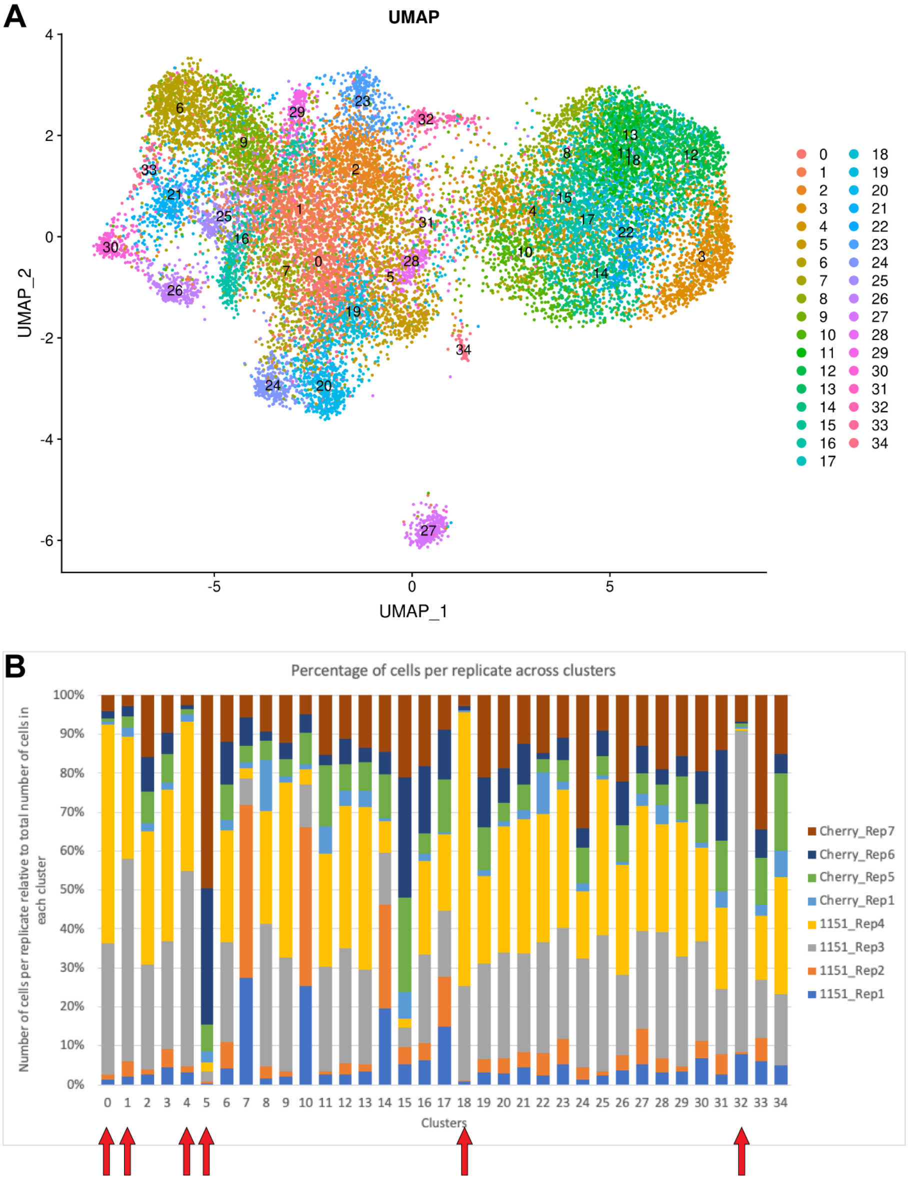

Appendix Figure S1. Clusters unevenly distributed across replicates. Related to Figure 1.

A. UMAP plot of the wild type dataset generated from wandering third instar larval wing discs prior to removal of unevenly represented clusters across replicates.

B. Percentage of cells in each replicate across clusters relative to the total number of cells per cluster. Four replicates *1151-GAL4* are indicated as 1151_Rep1, 1151_Rep2, 1151_Rep3, 1151_Rep4 and four replicates *1151>mCherry-RNAi* as Cherry_Rep1, Cherry_Rep5,

Cherry_Rep6, and Cherry_Rep7. Red arrows: clusters unevenly represented across replicates that were removed in downstream analysis.

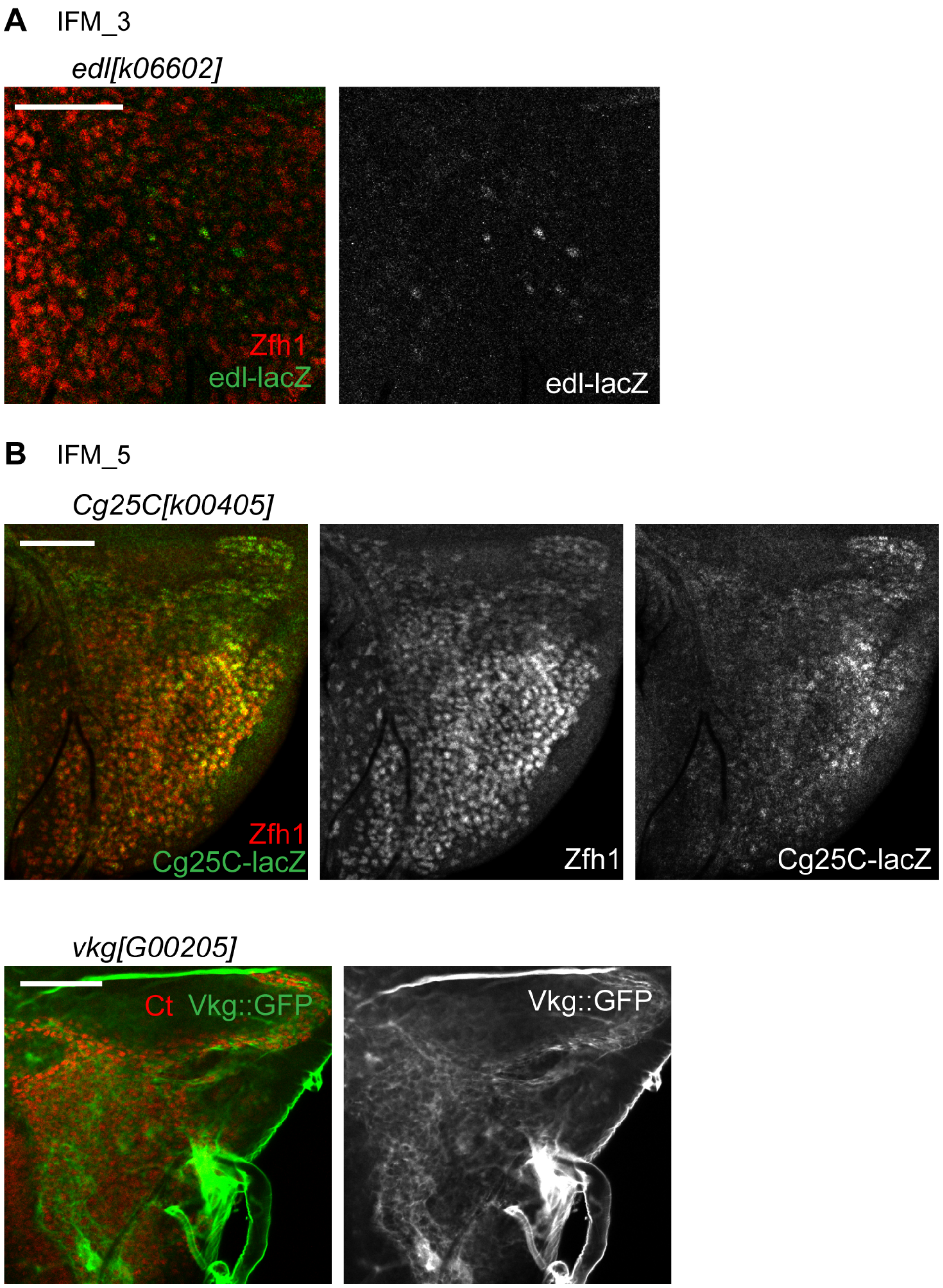

Appendix Figure S2. Spatial localization over the wing discs of gene markers for IFM clusters.

Related to Figure 5.

Confocal single plane images of third instar larval wing discs:

A. *edl[k06602]-lacZ* stained with anti-β-gal (green) and anti-Zfh1 (red).

B. *Cg25C[k00405]-lacZ* stained with anti-β-gal (green) and anti-Zfh1 (red) and *vgk[G00205]-GFP* (green) stained with anti-Ct (red).

Scale bars: 50 μm. Full genotypes: (A) *y-, w-; edl[k06602]; +*, (B) *y-, w-; Col4a1[k00405]-lacZ; +* and *w-*; *vkg-GFP[G00205]*;+.

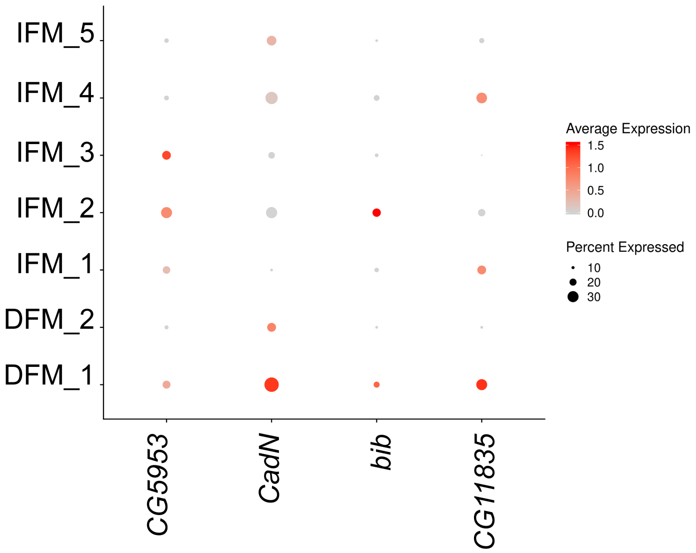

Appendix Figure S3. Additional gene markers for myoblast clusters. Related to Figure 6.

Dot plot showing the expression of the differentially expressed genes across myoblast clusters of the reference single cell atlas. Color intensity represents the average normalized expression level. Dot diameter represents the fraction of cells expressing each gene in each cluster.

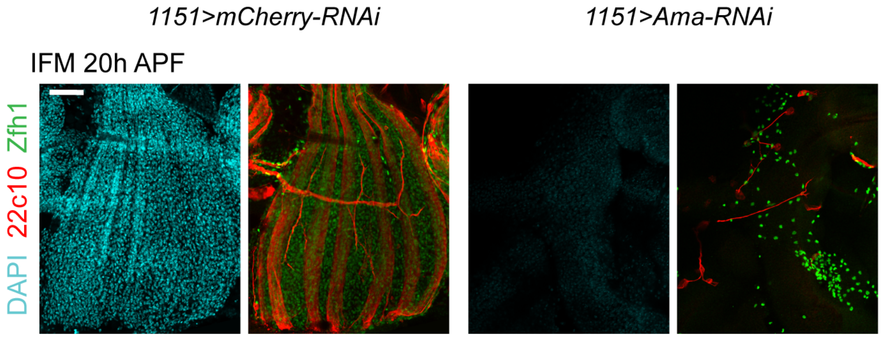

Appendix Figure S4. Loss of *Ama* in myoblasts blocks myoblast fusion in developing pupa.

Related to Figure 8.

Confocal single plane images of *1151>mCherry-RNAi* and *1151>Ama-RNAi* forming IFM (DLM) at 20 h APF stained with anti-Zfh1 (green), anti-Futsch/22c10 (red) and DAPI (cyan).

Scale bar: 50 µm. Anterior up. Full genotypes: *1151-GAL4; +; UAS-mCherry-RNAi* and *1151-GAL4; +; UAS-Ama[HMS00297]-RNAi.*

Appendix Tables

Gene	RNAi -Stock Number	Lethality	Flight defect	Insertion	Source
**Act57B**	31551	no	none	P{TRiP.JF01113}attP2	BDSC
**Alk**	27518	no	none	P{TRiP.JF02668}attP2	BDSC
**Ama**	33416	yes (early)	N/A	P{TRiP.HMS00297}attP2	BDSC
**Ama**	22944	yes (mild, early)	N/A	P{GD12733}v22944	VDRC
**Argk**	41697	no	none	P{TRiP.HMS02262}attP2	BDSC
**Argk**	31951	yes (pharate)	N/A	P{TRiP.JF02243}attP2	BDSC
**bib**	27691	no	none	P{TRiP.JF02771}attP2	BDSC
**CadN**	41982	no	none	P{TRiP.HMS02380}attP2	BDSC
**CadN**	27503	no	none	P{TRiP.JF02653}attP2	BDSC
**CG11835**	41725	no	flight defect	P{TRiP.HMS02291}attP2	BDSC
**CG5953**	57287	no	none	P{TRiP.HMJ21225}attP40	BDSC
**CG5953**	57543	no	none	P{TRiP.HMC04860}attP40	BDSC
**CG9650**	58323	no	none	P{TRiP.HMJ22453}attP40	BDSC
**CG9650**	26713	no	none	P{TRiP.JF02253}attP2	BDSC
**chinmo**	33638	yes (early)	N/A	P{TRiP.HMS00036}attP2	BDSC
**chinmo**	31738	yes (pupa)	N/A	P{TRiP.HM04048}attP2	BDSC
**chinmo**	62873	yes (early)	N/A	P{TRiP.HMC05346}attP40	BDSC
**chinmo**	26777	no	none	P{TRiP.JF02341}attP2	BDSC
**Col4a1**	44520	no	none	P{TRiP.HMC02910}attP2	BDSC
**con**	28967	no	none	P{TRiP.HM05178}attP2	BDSC
**E(spl)m3-HLH**	55302	no	none	P{TRiP.HMC03989}attP2	BDSC

**E(spl)m3-HLH**	25977	no	none	P{TRiP.JF0199 9}attP2	BDSC
**E(spl)m6-BFM**	66950	no	none	P{TRiP.HMS05 416}attP40	BDSC
**E(spl)m7-HLH**	29327	no	none	P{TRiP.JF0248 9}attP2	BDSC
**E(spl)malpha-BFM**	66949	no	none	P{TRiP.HMS05 415}attP40	BDSC
**E(spl)mdelta-HLH**	26203	no	mild flight defect	P{TRiP.JF0210 1}attP2	BDSC
**E(spl)mdelta-HLH**	65129	no	none	P{TRiP.HMC05 943}attP2	BDSC
**edl**	57552	no	none	P{TRiP.HMC04 869}attP40	BDSC
**eEF1alpha2**	64659	no	flight defect	P{TRiP.HMC05 694}attP40	BDSC
**h**	34326	no	none	P{TRiP.HMS01 313}attP2	BDSC
**hoip**	21752	no	none	P{GD11169}v2 1752	VDRC
**LamC**	31621	yes (early)	N/A	P{TRiP.JF0140 6}attP2	BDSC
**mamo**	60111	yes (early)	N/A	P{TRiP.HMC05 105}attP2	BDSC
**mamo**	26766	no	none	P{TRiP.JF0233 0}attP2	BDSC
**mamo**	51770	no	mild flight defect	P{TRiP.HMC03 325}attP40	BDSC
**mid**	50681	no	none	P{TRiP.HMC03 082}attP2	BDSC
**nkd**	67788	no	flight defect	P{TRiP.HMS05 702}attP40	BDSC
**nop5**	55262	yes (early)	N/A	P{TRiP.HMC03 949}attP40	BDSC
**Nrt**	28742	no	none	P{TRiP.JF0317 0}attP2	BDSC
**side**	50642	no	none	P{TRiP.HMC03 042}attP2	BDSC
**sp1**	35777	no	mild flight defect	P{TRiP.HMS01 526}attP2	BDSC
**SPARC**	40885	yes (early)	N/A	P{TRiP.HMS02 133}attP40	BDSC
**stg**	34831	mild (pharate)	flight defect	P{TRiP.HMS00 146}attP2	BDSC
**stg**	29556	no	none	P{TRiP.JF0323 5}attP2	BDSC

**Ten-a**	29439	no	none	P{TRiP.JF03375}attP2	BDSC
**vkg**	50895	yes (early)	N/A	P{TRiP.HMC02400}attP2	BDSC
**wb**	29559	no	none	P{TRiP.JF03238}attP2	BDSC

Appendix Table S1. Functional analysis of myoblast markers by screening muscle-related phenotype using *Mef2-GAL4* driver. Related to Figure 6A-B.

The *UAS-RNAi* lines were crossed to *Mef2-GAL4*. List of stocks and genes used in the screen.

Both viability and flight ability were assessed unless phenotype was lethal.

Gene	RNAi -Stock Number	Lethality	Flight defect	Insertion	Source
**Ama**	33416	yes (pharate)	NA	P{TRiP.HMS00297}attP2	BDSC
**SPARC**	40885	no	none	P{TRiP.HMS02133}attP40	BDSC
**chinmo**	33638	yes (pharate)	NA	P{TRiP.HMS00036}attP2	BDSC
**chinmo**	31738	Yes (mild, pharate)	NA	P{TRiP.HM04048}attP2	BDSC
**chinmo**	62873	no	NA	P{TRiP.HMC05346}attP40	BDSC
**nop5**	55262	yes (pharate)	N/A	P{TRiP.HMC03949}attP40	BDSC
**mamo**	60111	yes (early)	N/A	P{TRiP.HMC05105}attP2	BDSC
**LamC**	31621	no	none	P{TRiP.JF01406}attP2	BDSC
**vkg**	50895	no	none	P{TRiP.HMC02400}attP2	BDSC

Appendix Table S2. Functional analysis of myoblast markers by screening muscle-related phenotype using *1151-GAL4* driver. Related to Figure 6C-D.

The *UAS-RNAi* lines were crossed to *1151-GAL4* drivers if cross to *Mef2-GAL4* showed early lethality. List of stocks and genes used in the screen. Both viability and flight ability were assessed unless phenotype was lethal.

Gene	Stock	Type	Source	Insertion
**UAS-G-TRACE**		cell lineage system	Evans et al 2019, Nat Methods	P{UAS-RedStinger} 4, P{UAS-FLP1.D}JD1, pUbi-63E-STOP-Stinger insert 9F6
**UAS-Ama^{OE} (II)**		Overexpression (UAS-cDNA)	This work	UAS-Ama-FLAG-HA (DGRC 1621050)
	9723	PhiC31 integrase-mediated transgenesis	BDSC	PBac{y[+]-attP-3B}VK00002
**1151**		GAL4	Cripps	
**Mef2**	27390	GAL4	BDSC	P{GAL4-Mef2.R}3
**Ama**	103970	GAL4	KYOTO Stock Center (DGRC)	P{GawB}Ama[NP1297]
**SPARC**	77473	GAL4	BDSC	Mi{GT-GAL4}SPARC[MI00329-GAL4]
**E(spl)m3-HLH**	46517	GAL4	BDSC-Janelia	P{GMR10E12-GAL4}attP2
**E(spl)m6-BFM**		GAL4	Jagla (Aradhya et al. eLife 2015;4:e08497)	m6gap-GAL4
**E(spl)m6-BFM**		GFP reporter	Jagla (Aradhya et al. eLife 2015;4:e08497)	m6gapGFP
**E(spl)m7-HLH**	55839	GFP tag	BDSC	PBac{E(spl)m7-HLH-GFP.FPTB}VK00037
**E(spl)mbeta-HLH**	65294	GFP tag	BDSC	E(spl)mbeta-HLH-GFP.FPTB}attP40
**grh**	42272	GFP tag	BDSC	PBac{grh-GFP.FPTB}VK00033
**vkg**	110692	GFP tag	KYOTO Stock Center (DGRC)	vkg-GFP{PTT-un1}G00454
**vkg**	110626	GFP tag	KYOTO Stock Center (DGRC)	vkg-GFP{PTT-un1}G00205
**Argk**	51522	GFP trap	BDSC	P{PTT-GB}Argk[CB03789]
**nkd**	30664	GFP trap	BDSC	Mi{MIC}nkd[MI00209]
**Ten-a**	60541	GFP trap	BDSC	Mi{PT-GFSTF.2}Ten-a[MI04411-GFSTF.2]
**Col4a1/Cg25C**	499	lacZ	BDSC	P{lArB}Col4a1[A109.1F2]
**Col4a1/Cg25C**	10479	lacZ	BDSC	P{lacW}Col4a1[k00405]
**Dad**	10305	lacZ	BDSC	P{w[+mC]=lacW}Dad[j1E4]
**dpp**	12379	lacZ	BDSC	P{ry[+t7.2]=PZ}dpp[10638]
**edl**	10633	lacZ	BDSC	P{lacW}edl[k06602]

**edl**	102845	lacZ	KYOTO Stock Center (DGRC)	P{lacW}edl[k12907]
**twi**		lacZ	Cripps	
**kirre**	rp298	nlacZ	Cripps	
**UAS-GFP**		Reporter	Frolov	P{UAS-nGFP}2
**UAS-mCD8::GFP**	32185	Reporter	BDSC	P{10XUAS-IVS-mCD8::GFP}attP2
**UAS-mCD8.ChRFP**	27392	Reporter	BDSC	P{UAS-mCD8.ChRFP}3
**UAS-myrRFP**	7118	Reporter	BDSC	P{UAS-myr-mRFP}1
**Act57B**	31551	RNAi	BDSC	P{TRiP.JF01113}attP2
**Alk**	27518	RNAi	BDSC	P{TRiP.JF02668}attP2
**Ama**	33416	RNAi	BDSC	P{TRiP.HMS00297}attP2
**Ama**	22944	RNAi	VDRC	P{GD12733}v22944
**Argk**	41697	RNAi	BDSC	P{TRiP.HMS02262}attP2
**Argk**	31951	RNAi	BDSC	P{TRiP.JF02243}attP2
**bib**	27691	RNAi	BDSC	P{TRiP.JF02771}attP2
**CadN**	41982	RNAi	BDSC	P{TRiP.HMS02380}attP2
**CadN**	27503	RNAi	BDSC	P{TRiP.JF02653}attP2
**CG11835**	41725	RNAi	BDSC	P{TRiP.HMS02291}attP2
**CG5953**	57287	RNAi	BDSC	P{TRiP.HMJ21225}attP40
**CG5953**	57543	RNAi	BDSC	P{TRiP.HMC04860}attP40
**CG9650**	58323	RNAi	BDSC	P{TRiP.HMJ22453}attP40
**CG9650**	26713	RNAi	BDSC	P{TRiP.JF02253}attP2
**chinmo**	33638	RNAi	BDSC	P{TRiP.HMS00036}attP2
**chinmo**	31738	RNAi	BDSC	P{TRiP.HM04048}attP2
**chinmo**	62873	RNAi	BDSC	P{TRiP.HMC05346}attP40
**chinmo**	26777	RNAi	BDSC	P{TRiP.JF02341}attP2
**Col4a1/Cg25C**	44520	RNAi	BDSC	P{TRiP.HMC02910}attP2
**Con**	28967	RNAi	BDSC	P{TRiP.HM05178}attP2
**E(spl)m3-HLH**	55302	RNAi	BDSC	P{TRiP.HMC03989}attP2
**E(spl)m3-HLH**	25977	RNAi	BDSC	P{TRiP.JF01999}attP2
**E(spl)m6-BFM**	66950	RNAi	BDSC	P{TRiP.HMS05416}attP40
**E(spl)m7-HLH**	29327	RNAi	BDSC	P{TRiP.JF02489}attP2
**E(spl)malpha-BFM**	66949	RNAi	BDSC	P{TRiP.HMS05415}attP40
**E(spl)mdelta-HLH**	26203	RNAi	BDSC	P{TRiP.JF02101}attP2
**E(spl)mdelta-HLH**	65129	RNAi	BDSC	P{TRiP.HMC05943}attP2
**edl**	57552	RNAi	BDSC	P{TRiP.HMC04869}attP40

**eEF1alpha2**	64659	RNAi	BDSC	P{TRiP.HMC05694}attP40
**EGFP**	41559	RNAi	BDSC	P{VALIUM20-EGFP.shRNA.3}attP40
**h**	34326	RNAi	BDSC	P{TRiP.HMS01313}attP2
**hoip**	21752	RNAi	VDRC	P{GD11169}v21752
**LamC**	31621	RNAi	BDSC	P{TRiP.JF01406}attP2
**mamo**	26766	RNAi	BDSC	P{TRiP.JF02330}attP2
**mamo**	60111	RNAi	BDSC	P{TRiP.HMC05105}attP2
**mamo**	51770	RNAi	BDSC	P{TRiP.HMC03325}attP40
**mCherry**	35785	RNAi	BDSC	P{VALIUM20-mCherry}attP2
**mid**	50681	RNAi	BDSC	P{TRiP.HMC03082}attP2
**nkd**	67788	RNAi	BDSC	P{TRiP.HMS05702}attP40
**nop5**	55262	RNAi	BDSC	P{TRiP.HMC03949}attP40
**Nrt**	28742	RNAi	BDSC	P{TRiP.JF03170}attP2
**side**	50642	RNAi	BDSC	P{TRiP.HMC03042}attP2
**sp1**	35777	RNAi	BDSC	P{TRiP.HMS01526}attP2
**SPARC**	40885	RNAi	BDSC	P{TRiP.HMS02133}attP40
**stg**	34831	RNAi	BDSC	P{TRiP.HMS00146}attP2
**stg**	29556	RNAi	BDSC	P{TRiP.JF03235}attP2
**Ten-a**	29439	RNAi	BDSC	P{TRiP.JF03375}attP2
**vkg**	50895	RNAi	BDSC	P{TRiP.HMC02400}attP2
**wb**	29559	RNAi	BDSC	P{TRiP.JF03238}attP2
**attP2**	36303	wildtype/control	BDSC	P{CaryP}attP2

Appendix Table S3. List of fly stocks used in this work.

Ab/dye	host species	identifier	source	dilution
**Anti-cut**	mouse IgG1	2B10	DSHB	1/50
**Anti-GFP (FITC)**	goat	ab6662	abcam	1/500
**Anti-beta-gal**	mouse IgG1	40-1a	DSHB	1/200
**Anti-Zfh1**	rabbit		Ruth Lehmann (NYU Langone Medical Center, New York)	1/1000
**Anti-Kettin**	rat IgG1	MAC155	Babraham Institute	1/1000
**Anti-mCherry 594**	rat IgG2a	M11240	Life Technologies Corporation	1/750
**Anti-beta-PS-integrin**	mouse IgG2b	CF.6G11	DSHB	1/50
**Anti-futsch**	mouse IgG1	22C10	DSHB	1/30
**Anti-Neurotactin**	mouse IgG2a	BP 106	DSHB	1/100
**Anti-Lamin C**	mouse IgG1	LC28.26-s	DSHB	1/100
**Anti-Fasciclin III**	rabbit	7G10	DSHB	1/100
**Anti-Hairy**	mouse	1279	T. Orenic, UIC	1/4
**Anti-wingless**	mouse	4D4	DSHB	1/50
**Anti-seven up**	mouse	5B11	DSHB	1/20
**Anti-Notch Intracellular domain (NICD)**	mouse	C17.9C6	DSHB	1/100
**Anti- Notch Extracellular domain (NECD)**	mouse	458.2H	DSHB	1/100
**Anti-Phospho-Histone H3**	rabbit	06-570	Millipore	1/200
**Anti-Cleaved Drosophila Dcp-1**	rabbit	Asp216	Cell Signaling Technology	1/500
**Phalloidin-TRITC**		P1951	Sigma	1/1000
**Phalloidin-Atto 488**		49409	Sigma	1/1000
**4′,6-Diamidino-2-phenylindole dihydrochloride (DAPI)**		D8417	Sigma	1/1000
**Cy™3 AffiniPure Donkey Anti-Mouse IgG (H+L)**	donkey	715-165-151	Jackson Immunoresearch Laboratories	1/300
**Alexa Fluor® 647 AffiniPure Donkey Anti-Mouse IgG (H+L)**	donkey	715-605-151	Jackson Immunoresearch Laboratories	1/300
**Cy™5 AffiniPure Donkey Anti-Rabbit IgG (H+L)**	donkey	711-175-152	Jackson Immunoresearch Laboratories	1/300
**Cy3-AffiniPure Donkey Anti-Rabbit IgG (H+L)**	donkey	711-165-152	Jackson Immunoresearch Laboratories	1/300
**Alexa Fluor® 647 AffiniPure Donkey Anti-Rat IgG (H+L)**	donkey	712-605-153	Jackson Immunoresearch Laboratories	1/300
**Cy™3 AffiniPure Donkey Anti-Rat IgG (H+L)**	donkey	712-165-153	Jackson Immunoresearch Laboratories	1/300

Appendix Table S4. List of antibodies and dyes used in this work.
