# Supplementary material for: A cell atlas of adult muscle precursors uncovers early events in fibre‐type divergence in Drosophila
Source: EMBO Rep. 2020 Aug 19;21(10):e49555. doi: 10.15252/embr.201949555 (PMC7534622; doi:10.15252/embr.201949555)
Supplement: Supplementary file 2 — Expanded View Figures PDF [file EMBR-21-e49555-s002.pdf]

# Expanded View Figures

**Figure EV1. Single-cell atlas of the proximal wing imaginal disc. Related to Fig 1.**

A, B UMAP plot of the wild-type reference single-cell atlas generated from wandering third instar larval wing discs coloured by replicates (A) and split by replicates (B). Four replicates *1151-GAL4* are indicated as 1151 and 4 replicates *1151>mCherry-RNAi* as Cherry.

C, D Number of genes with at least 1 UMI count and number of UMI across all cells (left and right panel, respectively) grouped by replicates (C) and cell type (D).

E Total number of cells per sample in the reference atlas after applying filters and removing clusters that were unevenly represented across replicates.

F Dimensions of the data were reduced by PCA. Genes (rows) and cells (columns) were ordered by their PCA scores. Heatmap showing distribution of the 500 most extreme cells and 30 most extreme genes. PC1 separates epithelial cells from myoblast cells as indicated by the muscle-specific genes *Him* and *zfh1*, and the epithelial genes *Fas3* and *grh*.

G Confocal single plane images of wandering third instar larval wing discs and orthogonal view of *twi-lacZ* (β-gal, in red) and *grh::GFP* (green). Scale bars: 50 μm. Full genotype: *w-; twi-lacZ; grh[VK00033]::GFP*.

H Dot plot showing the expression of the main markers distinguishing Trachea_1 from Trachea_2 across all clusters of the reference atlas.


© 2020 The Authors

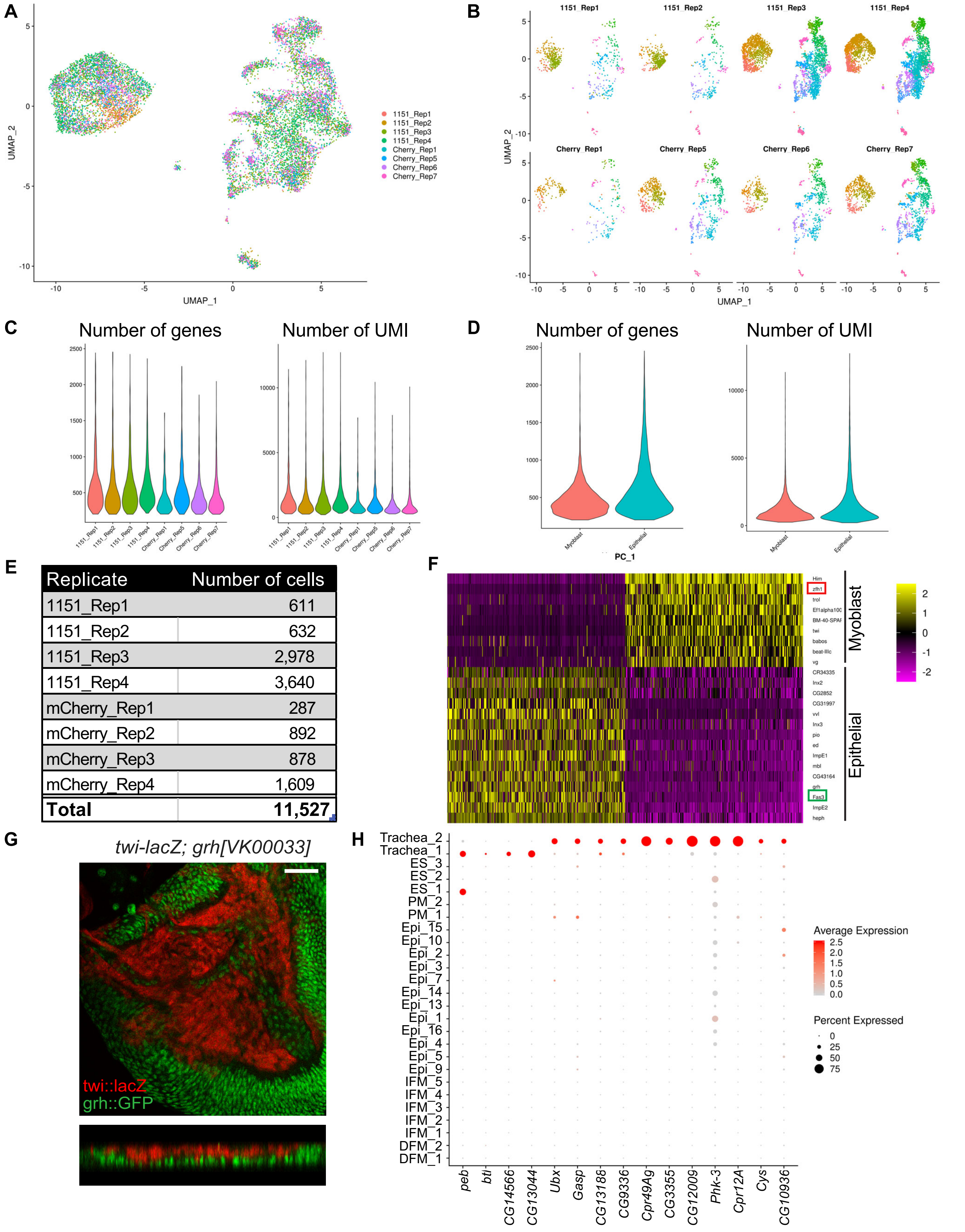


| Replicate | Number of cells |
|---|---|
| 1151_Rep1 | 611 |
| 1151_Rep2 | 632 |
| 1151_Rep3 | 2,978 |
| 1151_Rep4 | 3,640 |
| mCherry_Rep1 | 287 |
| mCherry_Rep2 | 892 |
| mCherry_Rep3 | 878 |
| mCherry_Rep4 | 1,609 |
| **Total** | **11,527** |

**Figure EV1.**

© 2020 The Authors

**Figure EV2. Markers for the epithelial clusters. Related to Fig 2.**

A Average expression of the genes used as known markers to map localization of epithelial clusters to the cell fate map of the wing discs in the reference atlas dataset. Marker gene for Epi_5 and 9 is *CG17278*, Epi_13 is *caup*, *ara*, *mirr* and *vn*, Epi_2 and 3 is *toe*, Epi_10 is *knrl*, Epi_15 is *tup*, and PM_2 is *Antp*.

B Violin plot representing the distribution of expression levels of *scabrous* (*sca*) across the epithelial clusters assigned as in the reference atlas dataset.

 © 2020 The Authors

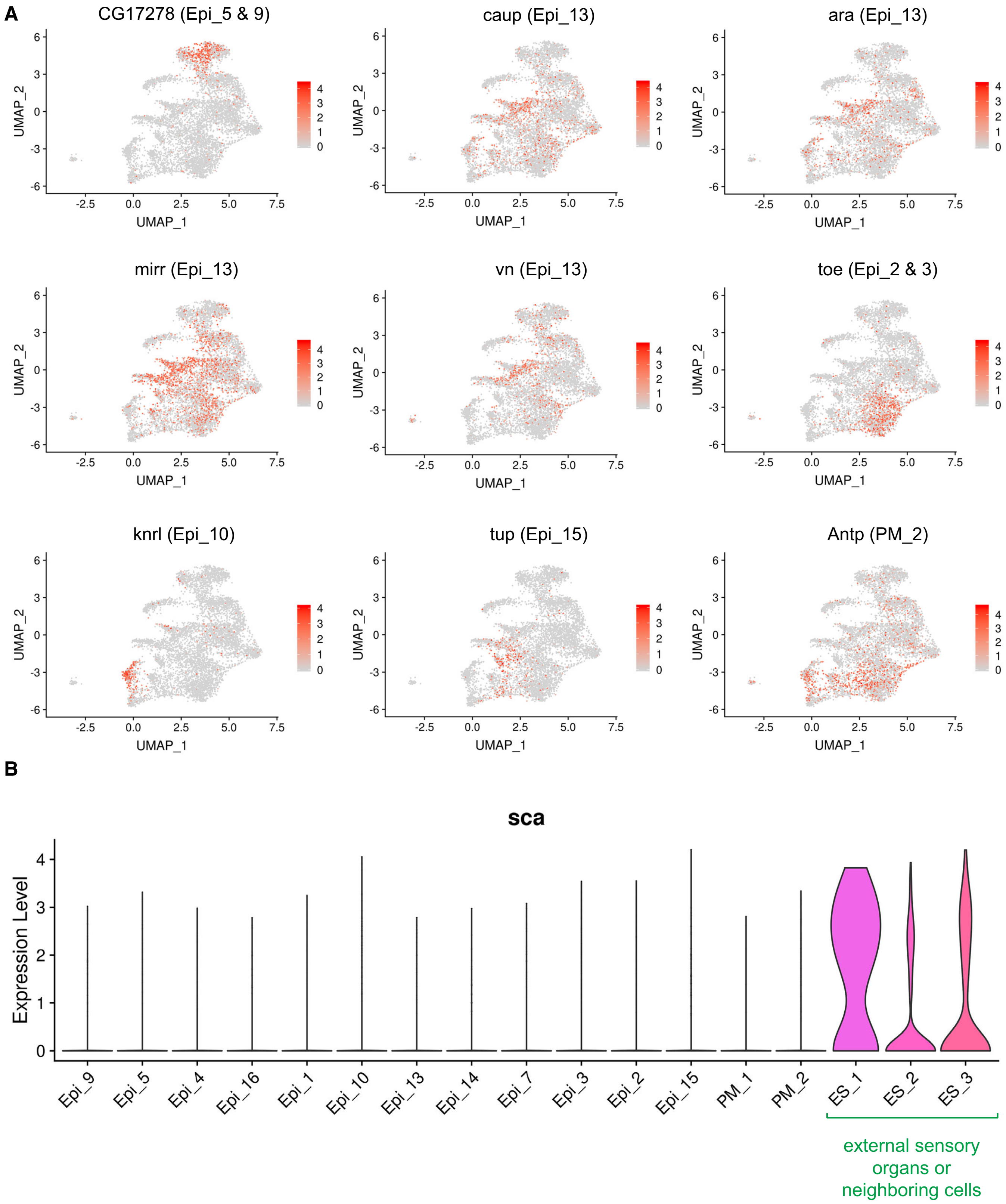
A
CG17278 (Epi_5 & 9)
caup (Epi_13)
ara (Epi_13)
mirr (Epi_13)
vn (Epi_13)
toe (Epi_2 & 3)
knrl (Epi_10)
tup (Epi_15)
Antp (PM_2)
UMAP_1
UMAP_2
B
sca
Expression Level
Epi_9
Epi_5
Epi_4
Epi_16
Epi_1
Epi_10
Epi_13
Epi_14
Epi_7
Epi_3
Epi_2
Epi_15
PM_1
PM_2
ES_1
ES_2
ES_3
external sensory organs or neighboring cells

**Figure EV2.**

**Figure EV3. Markers for DFM and IFM myoblast clusters. Related to Fig 3.**

A Heatmap showing the 10 tops differentially expressed gene markers for each myoblast cluster of the reference single-cell atlas dataset. Cells (columns) are clustered by the expression of the main marker genes (rows).

B Confocal single plane images of third instar larval wing discs stained with anti-Nrt (white) in DFM myoblast area of *Ama[NP1297]>ChFP* and *Argk[CB3789]::GFP*, top and bottom panel, respectively.

C–E Confocal single plane images of *Ama[NP1297]>gTRACE* showing the lineage of *Ama-GAL4* (green) and the active *GAL4* (red) in: (C) third instar larval wing disc stained with anti-Ct (white). Dashed yellow line: DFM myoblast area. Yellow arrowhead: cell lineage expression of *Ama* in few IFM myoblasts; (D) forming DFM at 28 h APF stained with anti-Zfh1 (white). White arrows: wing hinge; (E) adult IFM (specifically, DLM) stained with DAPI (white), anterior up.

Data information: Scale bars: 50 μm. Full genotypes: (B) *w-; +; Ama[NP1297]-GAL4/UAS-mCD8. ChRFP* and *y-, w-; +; Argk[CB03789]::GFP.* (C) *w-; UAS-gTRACE/+; Ama [NP1297]-GAL4/+.*

 © 2020 The Authors

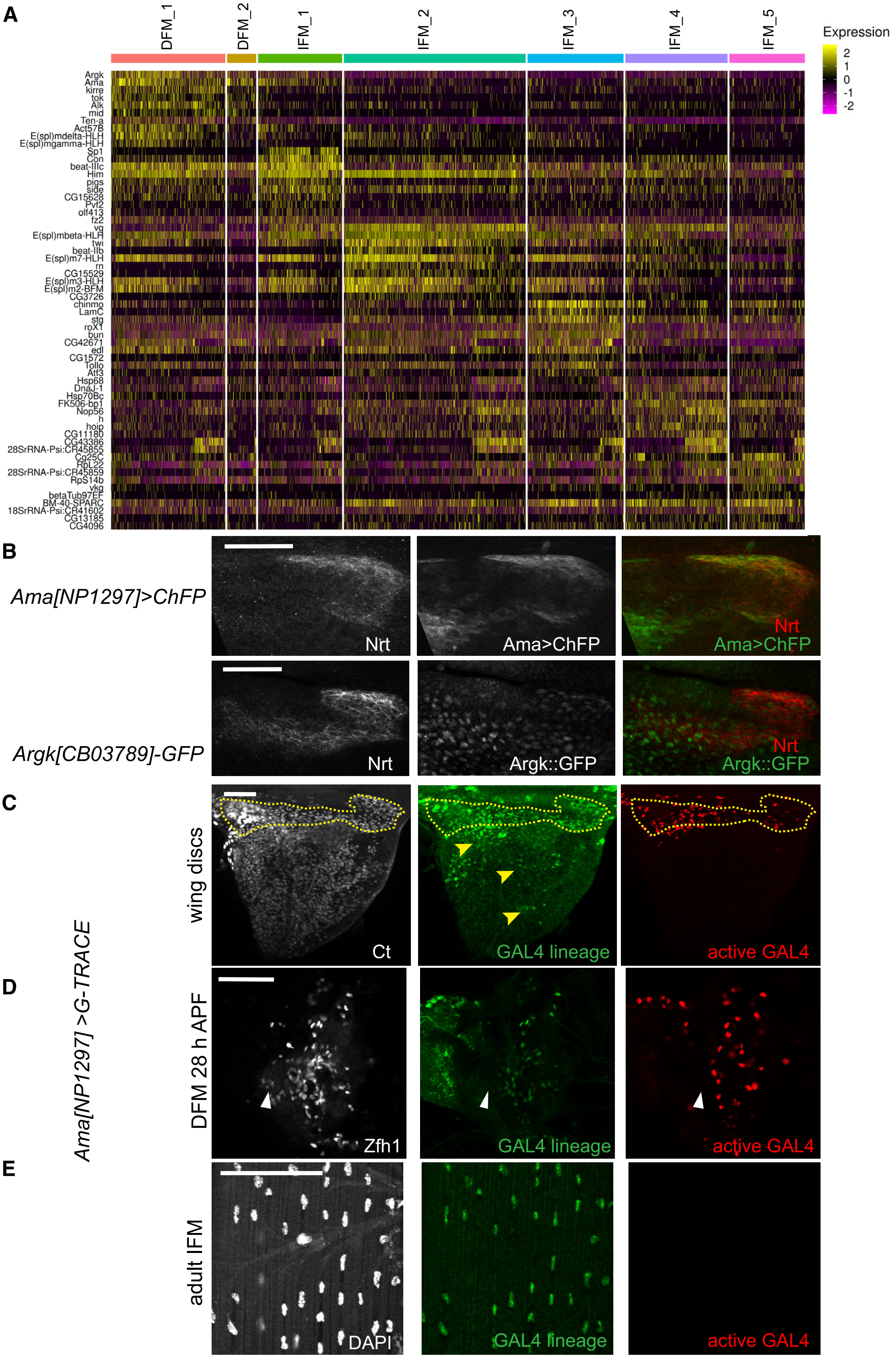
A
DFM_1
DFM_2
IFM_1
IFM_2
IFM_3
IFM_4
IFM_5
Expression
2
1
0
-1
-2
Argk
Ama
kirre
tok
Alk
mid
Ten-a
Act57B
E(spl)mdelta-HLH
E(spl)mgamma-HLH
Sp1
Con
beat-IIIc
Him
pigs
side
CG15628
Pvf2
olf413
fz2
vg
E(spl)mbeta-HLH
twi
beat-IIb
E(spl)m7-HLH
rn
CG15529
E(spl)m3-HLH
E(spl)m2-BFM
CG3726
chinmo
LamC
stg
roX1
bun
CG42671
edl
CG1572
Tollo
Atf3
Hsp68
DnaJ-1
Hsp70Bc
FK506-bp1
Nop56
h
hoip
CG11180
CG43386
28SrRNA-Psi:CR45855
Cg25C
RpL22
28SrRNA-Psi:CR45859
RpS14b
vkg
betaTub97EF
BM-40-SPARC
18SrRNA-Psi:CR41602
CG13185
CG4096
B
Ama[NP1297]>ChFP
Nrt
Ama>ChFP
Nrt
Ama>ChFP
Argk[CB03789]-GFP
Nrt
Argk::GFP
Nrt
Argk::GFP
C
wing discs
Ct
GAL4 lineage
active GAL4
D
Ama[NP1297] >G-TRACE
DFM 28 h APF
Zfh1
GAL4 lineage
active GAL4
E
adult IFM
DAPI
GAL4 lineage
active GAL4

**Figure EV3.**

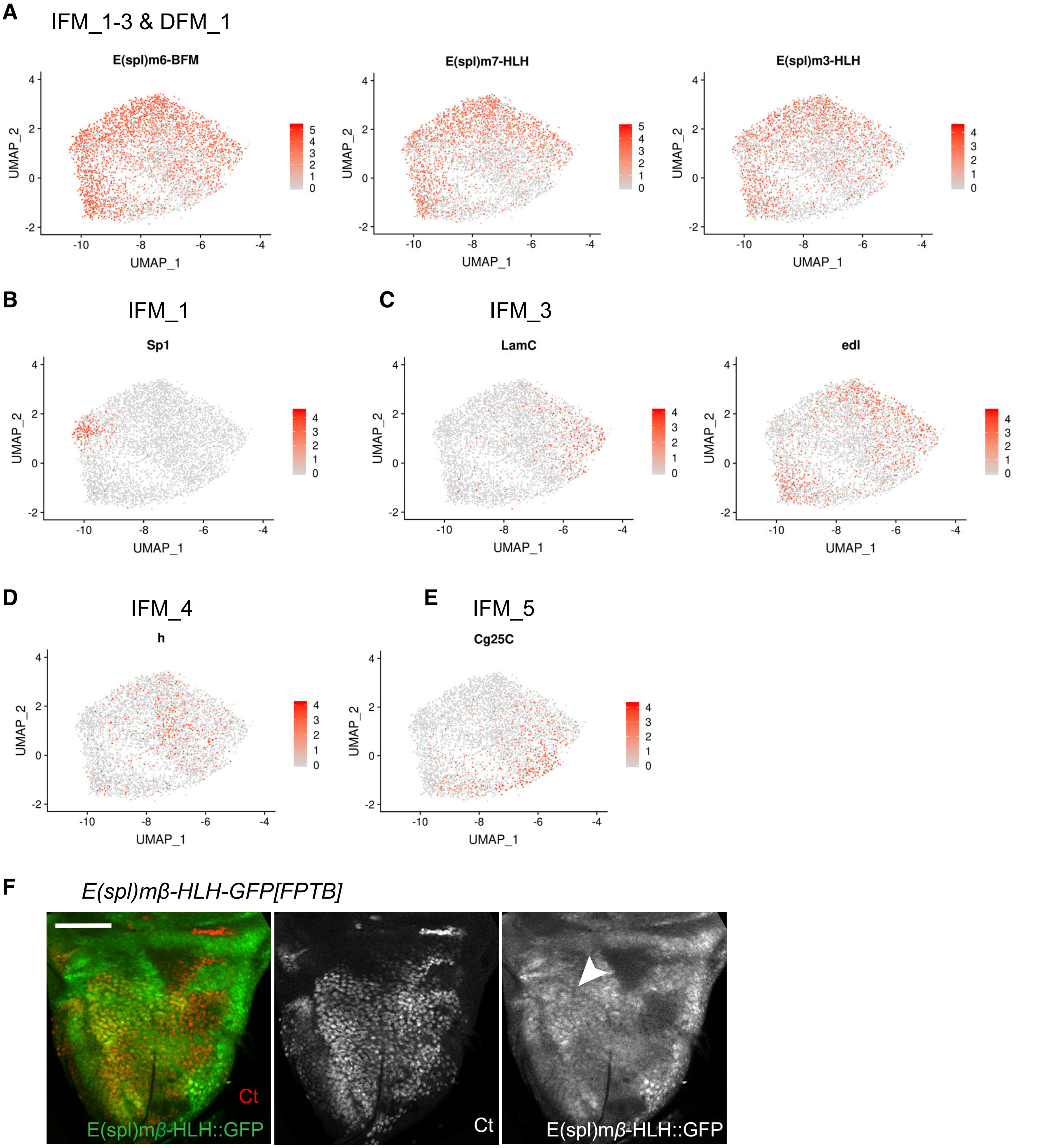


**Figure EV4. Markers for different state of differentiation. Related to Fig 4.**

A–E Average expression level of the genes identified as markers for the myoblast clusters assigned as in the reference atlas dataset. Marker gene for (A) IFM_1-3 and DFM_1 are *E(spl)-m6-BFM*, *E(spl)-m7-HLH*, *E(spl)-m3-HLH*, (B) IFM_1 is *Sp1*, (C) IFM_3 are *LamC* and *edl*., (D) IFM_4 is *h* and (E) IFM_5 is *Cg25C*.

F Confocal single plane image of third instar larval wing discs *E(spl)-mbeta-HLH-GFP[FPTB]* stained with anti-Ct (red). Scale bar: 50 μm. Full genotype: *y- ω-*; P{*E(spl) mbeta-HLH-GFP.FPTB*}*attP40*.

Data information: White arrowheads point to location of IFM_1-2 cells.


© 2020 The Authors

**Figure EV5. Loss of Amalgam in myoblast of larval wing disc. Related to Fig 7.**

A UMAP plot of *1151>Ama-RNAi* from third instar larval wing discs coloured by cell type (myoblast, epithelial and tracheal cells) and split by replicate (Rep1, Rep4 and Rep5). The total number of cells after applying filters was 1,232, 706 and 1,400 for each replicate, respectively.

B The cells from *1151>Ama-RNAi* dataset were projected into the reference single-cell atlas. Number of cells in each cluster normalized to the total number of epithelial cells in each genotype is plotted across epithelial cells.

C Violin plot representing the distribution of expression levels of the epithelial marker *grh* across all the clusters, split by *1151>Ama-RNAi* (pink) and reference cell atlas dataset (blue).

D, E Confocal single plane images of third instar larval wing discs from *1151>mCherry-RNAi* and *1151>Ama-RNAi* stained with anti-Ct (green), DAPI (red) and (D) anti-pH3 (magenta) or (E) anti-Dcp1 (magenta).

F Dot plots showing the expression levels of the known differentiation genes across the cluster of myoblast cells in *1151>Ama-RNAi* and the reference cell atlas.

Data information: Scale bars: 50 μm. Full genotypes: *1151-GAL4; +; UAS-mCherry-RNAi* and *1151-GAL4; +; UAS-Ama[HMS00297]-RNAi*.

© 2020 The Authors

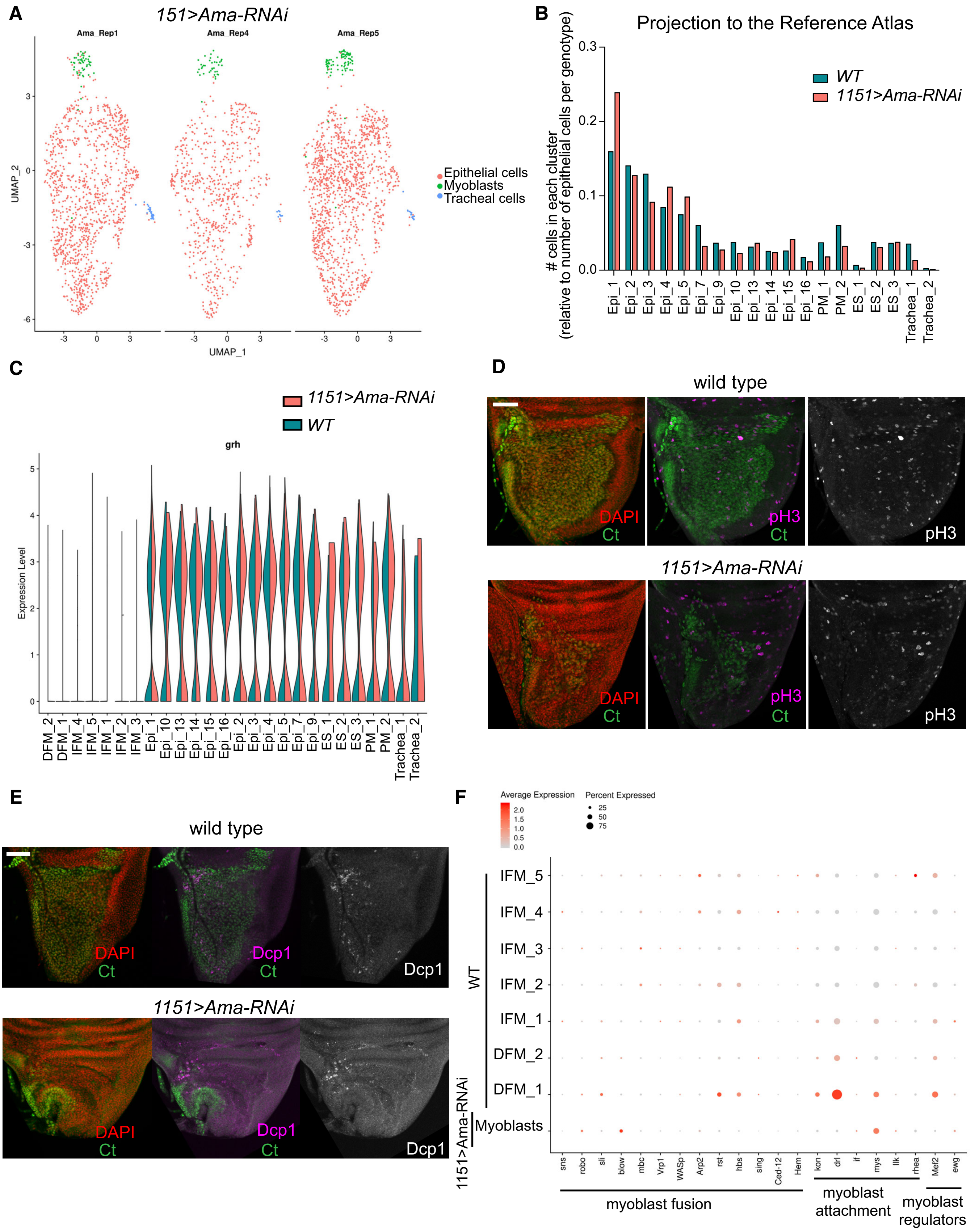


**Figure EV5.**


© 2020 The Authors
