# Supplementary figures and images for: A cell atlas of adult muscle precursors uncovers early events in fibre‐type divergence in Drosophila
Source: EMBO Rep. 2020 Aug 19;21(10):e49555. doi: 10.15252/embr.201949555 (PMC7534622; doi:10.15252/embr.201949555)

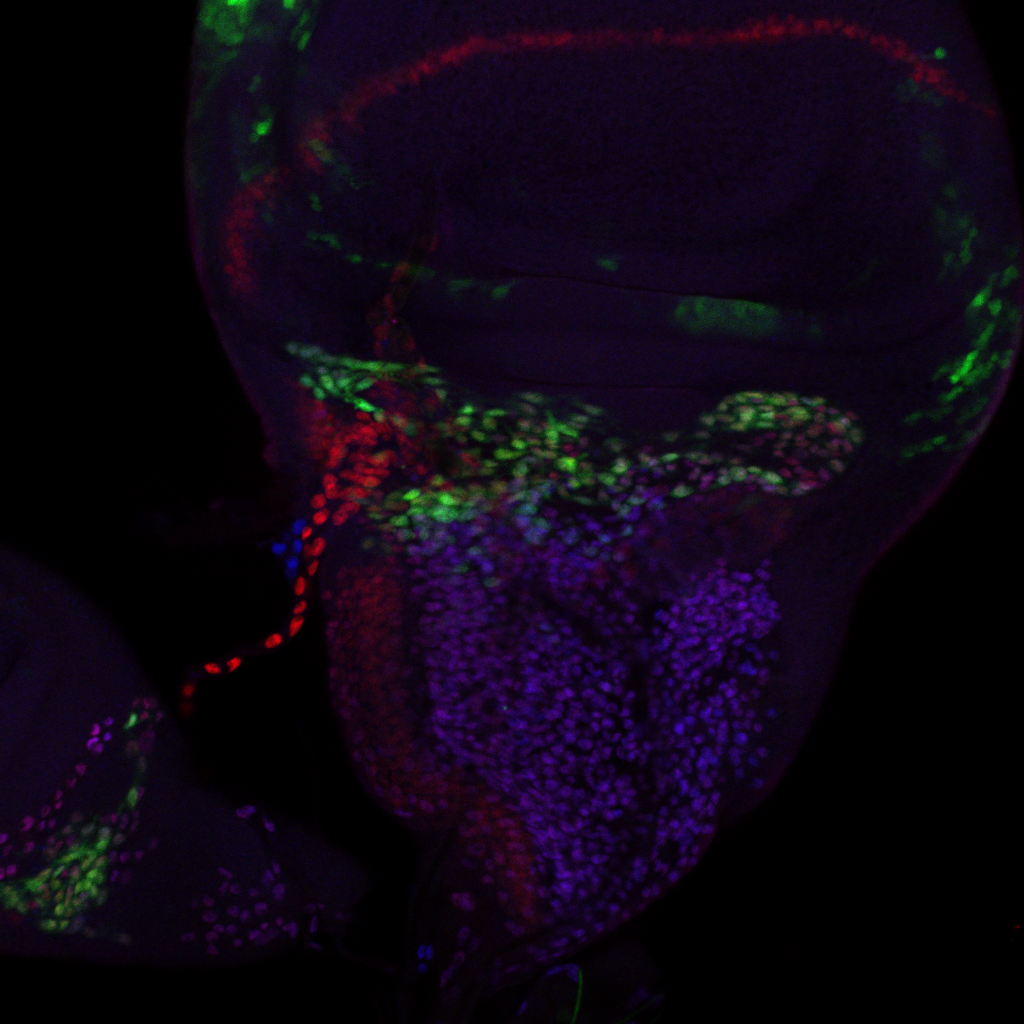

Supplement: Supplementary file 12 — Source Data for Figure 3 [file EMBR-21-e49555-s011.zip › EMBOR-2019-49555-T_SourceDataForFigure3I-wing disc AmaGAL4-GFP ct-red zfh1-blue 20x.tif]

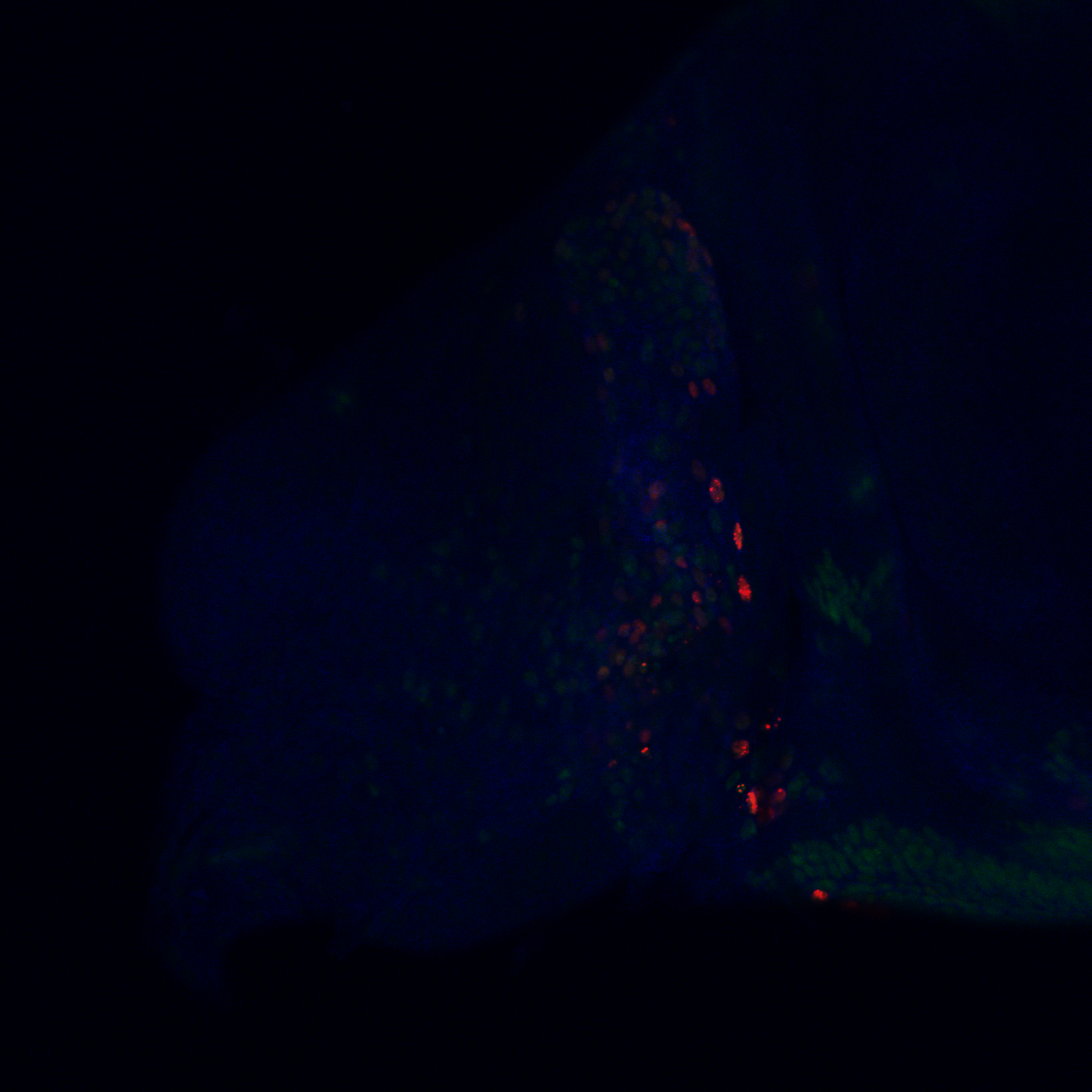

Supplement: Supplementary file 12 — Source Data for Figure 3 [file EMBR-21-e49555-s011.zip › EMBOR-2019-49555-T_SourceDataForFigure3K-wing disc AmaGAL4-gtrace RNA Ama-blue 20x.tif]

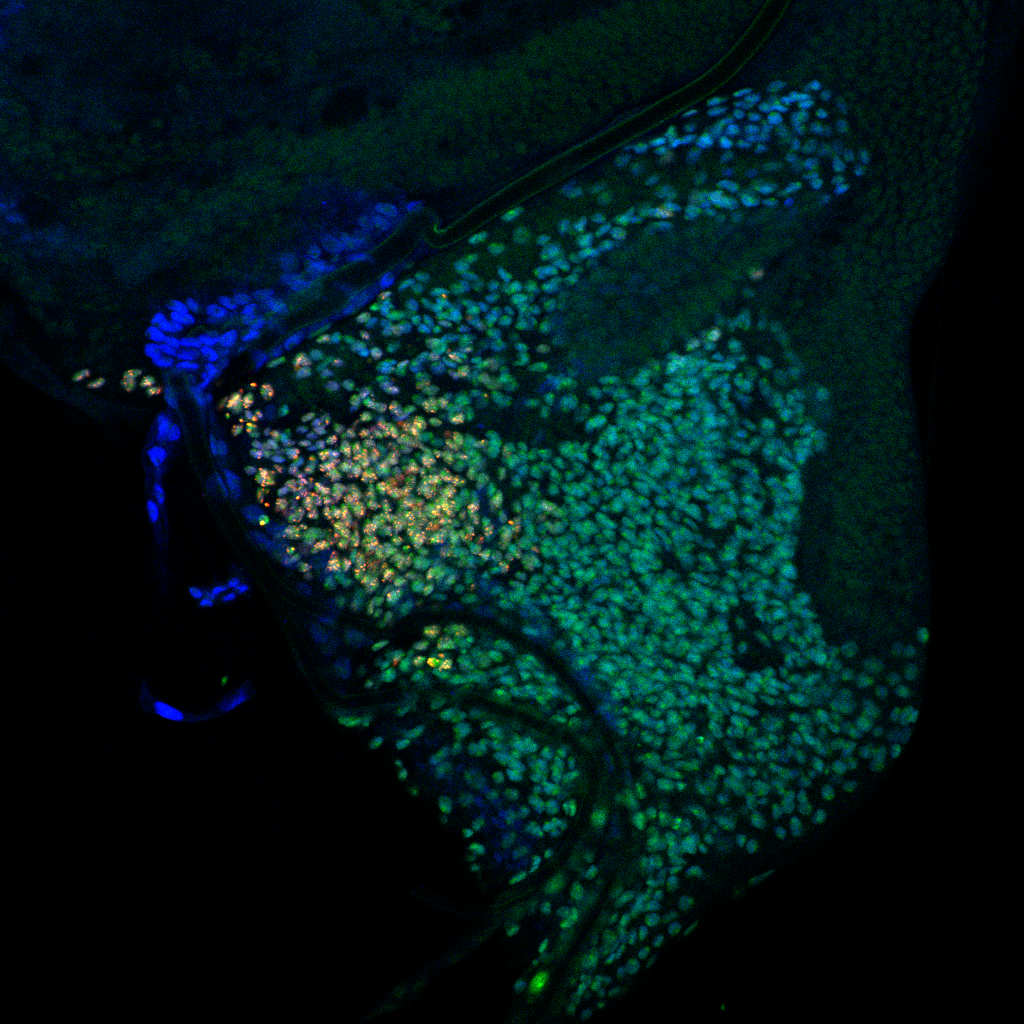

Supplement: Supplementary file 13 — Source Data for Figure 4 [file EMBR-21-e49555-s012.zip › EMBOR-2019-49555-T_SourceDataForFigure4I-wingdisc m6-GAL4-GTRACE cut-blue 40x.tif]

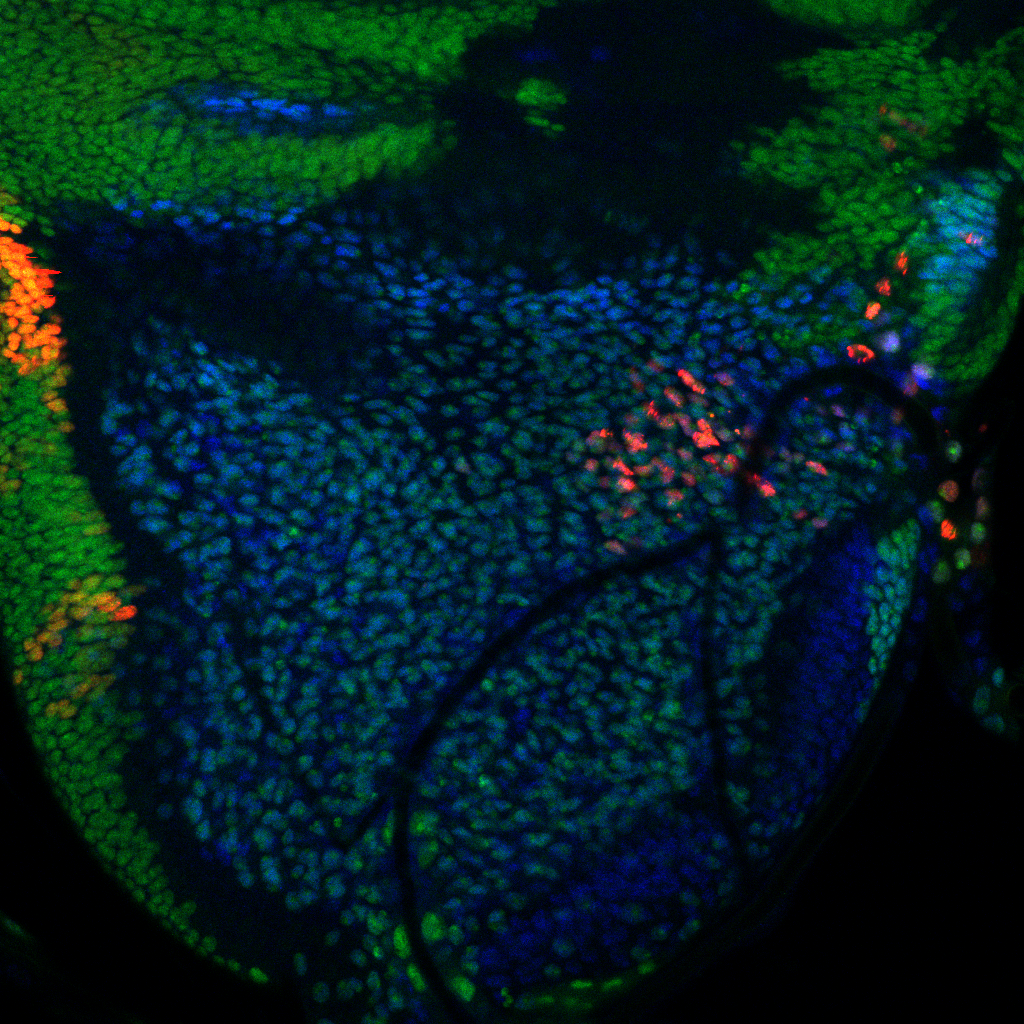

Supplement: Supplementary file 13 — Source Data for Figure 4 [file EMBR-21-e49555-s012.zip › EMBOR-2019-49555-T_SourceDataForFigure4H-wingdisc m3-GAL4 BL46517 GTRACE cut-blue 40x.tif]

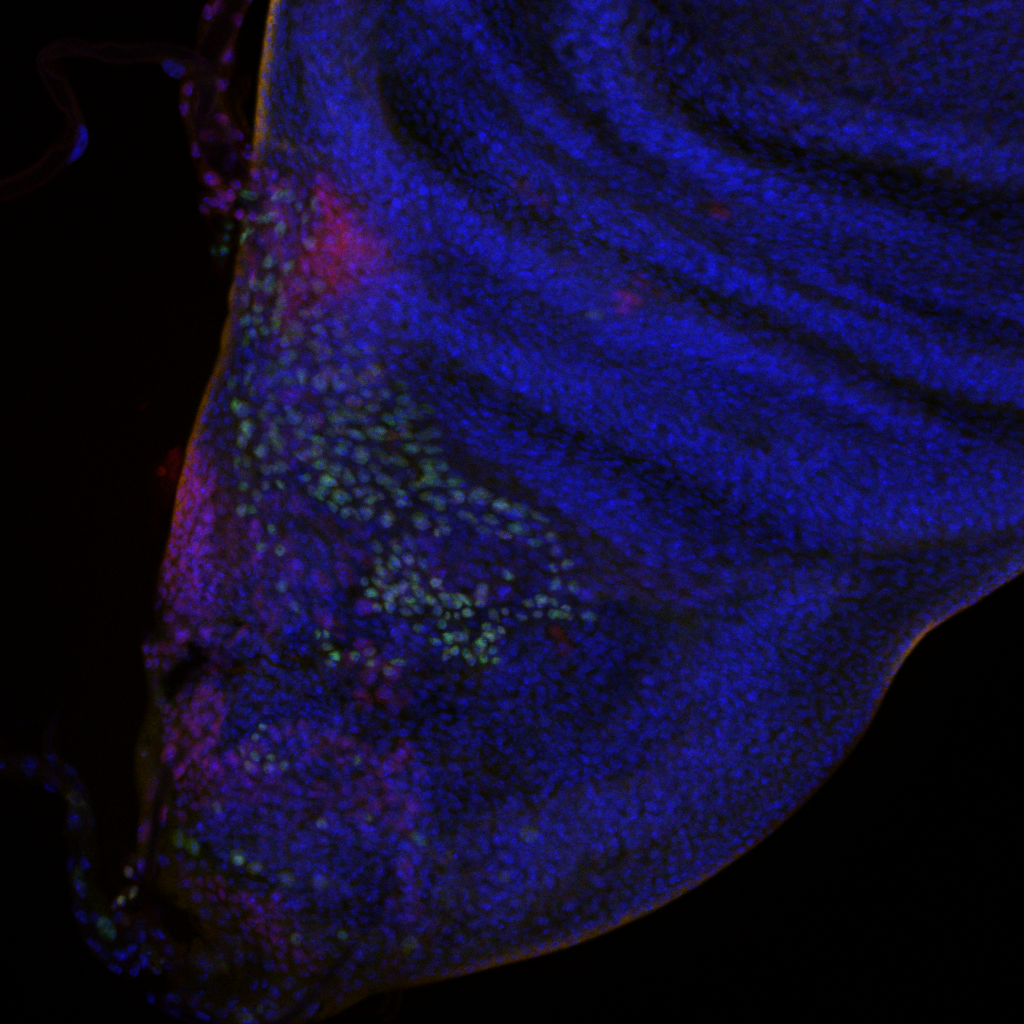

Supplement: Supplementary file 15 — Source Data for Figure 7 [file EMBR-21-e49555-s014.zip › EMBOR-2019-49555-T_SourceDataForFigure7D-wing disc 1151-AmaRNAi DAPI ct-red zfh1-green 20x.tif]

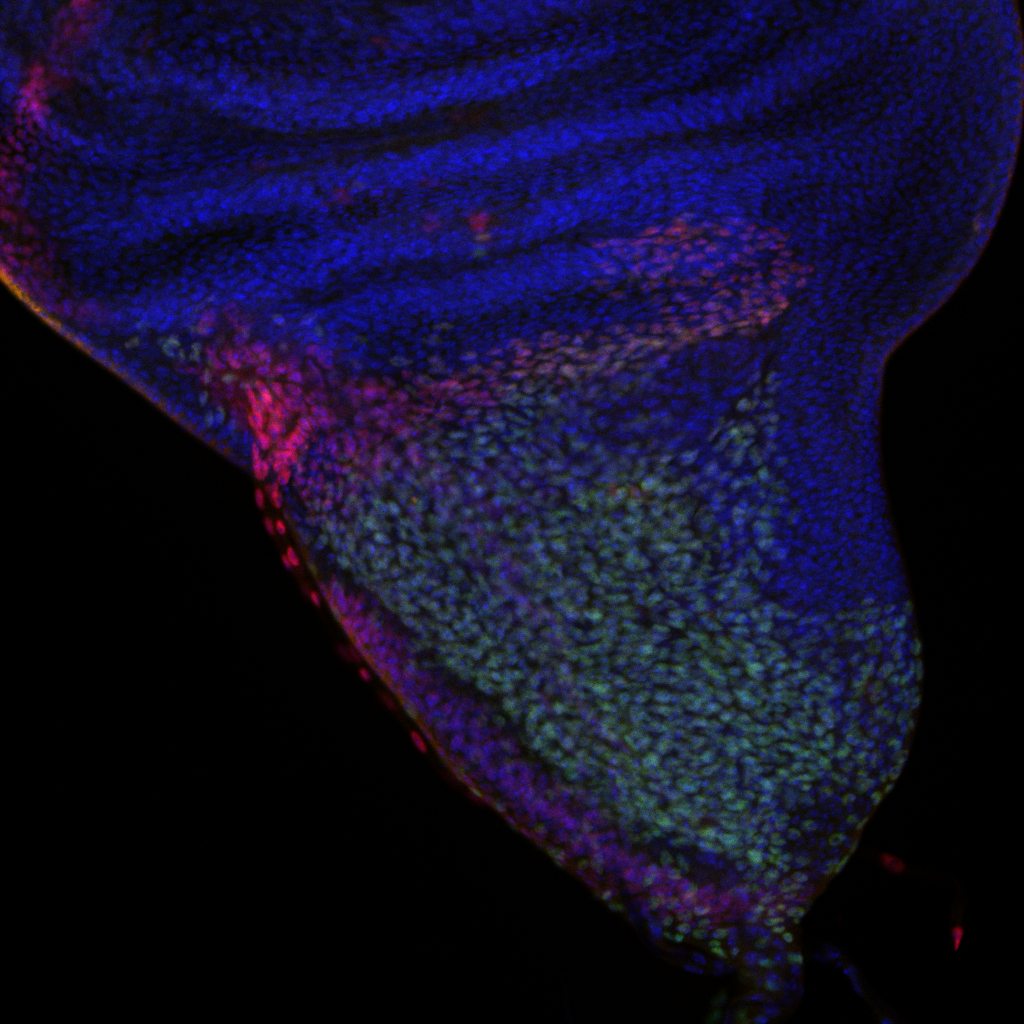

Supplement: Supplementary file 15 — Source Data for Figure 7 [file EMBR-21-e49555-s014.zip › EMBOR-2019-49555-T_SourceDataForFigure7D-wing disc 1151-mCherryRNAi DAPI ct-red zfh1-green 20x.tif]
